# ALBI grade for outcome prediction in patients affected by hepatocellular carcinoma treated with transarterial radioembolization

**DOI:** 10.3389/fnume.2022.934446

**Published:** 2022-07-22

**Authors:** Fabrizia Gelardi, Marcello Rodari, Cristiano Pini, Roberta Zanca, Alessia Artesani, Giovanni Tosi, Arturo Chiti, Martina Sollini

**Affiliations:** ^1^Department of Biomedical Sciences, Humanitas University, Milan, Italy; ^2^Istituti di Ricovero e Cura a Carattere Scientifico (IRCCS) Humanitas Research Hospital, Milan, Italy

**Keywords:** ALBI (albumin-bilirubin) score, transarterial radioembolization TARE, HCC, radionuclide therapy, prognosis

## Abstract

**Introduction and aim:**

Diagnosis of hepatocellular carcinoma (HCC) often occurs when the disease is unresectable and therapeutic options are limited. The extent of disease and liver function according to Child-Pugh (C-P) classification are the main prognostic factors guiding clinicians in the management of HCC. The integration of albumin-bilirubin (ALBI) grade is emerging to assess liver function on account of its objectivity and reproducibility. Our aim was to investigate the value of the ALBI grade in predicting the outcome in patients treated with transarterial radioembolization (TARE).

**Methods:**

We retrospectively enrolled patients with advanced and unresectable HCC treated with TARE in our institution. All patients underwent a preliminary dosimetric study before Yttrium-90 resin microsphere TARE. Barcelona Clinic Liver Cancer (BCLC), C-P, and ALBI scores were established at the time of TARE. Overall survival (OS), progression-free survival (PFS), and survival after TARE were assessed with the Kaplan-Meier method. Survival analyses were stratified according to ALBI grade, C-P, and BCLC classification. Univariate and multivariate Cox proportional regression models determined the association between prognostic factors and clinical outcomes.

**Results:**

In total, 72 patients were included in the study, showing an OS of 51 months. The ALBI grade identified groups of patients with different prognoses both in the whole cohort and within the C-P classes, especially between ALBI 1 and ALBI 2. This result is confirmed also within BCLC classes. In treatment naïve patients, the ALBI grade was not able to predict outcomes, whereas the presence and degree of portal vein thrombosis (PVT) significantly affected prognosis.

**Conclusions:**

The ALBI grade provided a more accurate prognostic stratification than the C-P classification in patients with intermediate and advanced HCC treated with TARE. However, the outcome of HCC is affected not only by liver function but also by disease-related characteristics, such as disease burden and degree of PVT. Including the ALBI grade in clinical guidelines may improve the management of patients affected by HCC.

## Background and aim

Hepatocellular carcinoma (HCC) accounts for 90% of primary liver cancer and cirrhosis resulting from chronic hepatitis is the major risk factor (1). HCC occurs more frequently in men with an overall age-standardized male-to-female ratio of 3.55 (2). Unfortunately, HCC is mostly diagnosed at intermediate and advanced stages when the disease is unresectable and burdened by a poor prognosis (3, 4). European Association for the Study of the Liver (EASL) guidelines address a treatment strategy by tumor extent, liver function, and performance status, according to the Barcelona Clinic Liver Cancer (BCLC) classification (5). Treatments of the unresectable disease include—among others—transarterial radioembolization (TARE), which is recommended in case of ineffectiveness or contraindication to other endo-arterial treatments, even in presence of portal vein thrombosis (PVT) (5–7). Liver function and tumor burden are the two factors considered in clinical decision-making algorithms as, together with malignancy burden, they affect the outcome of HCC (8, 9). The Child-Pugh (C-P) classification is the most widely used system to assess liver function (10). C-P score is based on highly subjective clinical parameters, while some variables are interrelated (i.e., albumin and ascites) and therefore counted two times. In addition, objective laboratory values are simplified to categorical values, resulting in less informative (9, 11). C-P classification is not always accurate and its usefulness in prognostic stratification of HCC is limited, as most of the patients are placed in class A at the onset of disease (9). Moreover, patients with preserved liver function may be incorrectly assigned to C-P class B, although they would benefit from curative surgery. On the other hand, patients with liver impairment should be treated with less invasive ablative therapies or systemic agents, despite they were included in C-P A (12, 13). More recently, Johnson et al. proposed the integration of albumin-bilirubin (ALBI) grade as a new prognostic score to assess liver function (14). The ALBI grade considers only albumin and total bilirubin as continuous variables, overcoming the inherent limitations of C-P classification (9, 14). This new model is promising and could replace C-P as an inclusion criterion in the design of clinical trials on account of its objectivity and reproducibility (9). The aim of our study was to assess the role of the ALBI classification system in predicting outcomes in patients treated with TARE. We further investigated the impact of the independent disease- and treatment-related factors on prognosis.

## Methods

### Patients' selection

This was a single-center observational retrospective study. We enrolled consecutive patients with advanced and unresectable HCC treated with TARE in IRCCS Humanitas Research Hospital between March 2014 and September 2020. The liver multidisciplinary tumor board selected patients to be treated with TARE. The institutional ethics committee approved the study, which was conducted in accordance with the Declaration of Helsinki. All patients signed written informed consent before treatment according to good clinical practice. A specific written consent to participate was waived due to the observational and retrospective nature of the study.

### Treatment

Before treatment, all patients performed a dosimetric study with 99mTc-macroaggregated albumin (MAA) single-photon emission computerized tomography (SPECT)/CT to predict tumor and organs at risk (OAR) absorbed dose using the partition model method (15). The percentage of the systemic shunt was assessed and patients with significant shunts were excluded from treatment. To prevent the spread of resin microspheres to other organs, when necessary, a selective prophylactic embolization of distal hepatoenteric vessels visualized at preliminary arteriography was performed. All patients were treated with Yttrium-90 resin microspheres (SIR-Spheres, Sirtex Medical Pty Ltd., North Sydney, NSW, Australia). Yttrium-90 resin microspheres were administered by an experienced nuclear medicine physician (MR) in collaboration with interventional radiologists and medical physicists under local anesthesia. After treatment, patients underwent a PET/CT scan to confirm the placement of resin microspheres.

### Data collection

Clinical data were retrospectively collected from electronic medical records, such as patients' characteristics (sex and age), underlining cirrhosis etiology, previous treatments, and HCC characteristics (location, number of lesions, and presence/grade of PVT). PVT grade was classified according to Xu classification in type A in case of involvement of the main portal vein or both right and left branches of the portal vein and type B in case of involvement of either right or left branch of the portal vein (16). BCLC and C-P classes were calculated at the time of TARE. Treatment details were collected, such as the number of procedures, the treated lesions, the volume of the treated lesion(s), Yttrium-90 administered activity, tumor absorbed dose, and percentage of a systemic shunt. Values of routine laboratory tests (albumin, total bilirubin, international normalized ratio (INR), alkaline phosphatase, and alpha-fetoprotein [AFP]) before the procedure were recorded. ALBI score was calculated by using values of albumin and total bilirubin assayed before TARE. ALBI score ≤-2.60 was defined as grade 1, >-2.60 but ≤-1.39 as grade 2, and >-1.39 as grade 3 (14).

### Follow-up and outcome assessment

Treatment response was clinically and radiologically assessed. All patients performed laboratory tests and clinical examinations about 15 days after the procedure. Radiological imaging (CT or MRI) was performed 45 days and 3 months after TARE. Subsequently, patients performed routine follow-up visits, laboratory tests, and radiological imaging. Overall Survival (OS) was established from the date of diagnosis to the date of death. A 4-month survival time was assumed in patients with disease progression and forwarded to Integrated Domiciliary Assistance (ADI) at the last follow-up (5, 17). Patients lost at follow-up were contacted by telephone. Still-living patients were censored at the last follow-up. Progression-free survival (PFS) was established from the date of TARE to the date of clinical or radiological disease progression. Survival after TARE was calculated from the time of the first TARE to the date of death. Follow-up data collection ended on 1 March 2022.

### Statistical analyses

Baseline patients' characteristics were summarized in frequency tables. Non-normally distributed continuous variables were reported as the median and interquartile range (IQR). Categorical variables were reported as the number of cases and percentages. Treatment-related data were aggregated in patients who underwent more than one procedure (i.e., volume of treated lesions, administered activity, tumor absorbed dose, and pulmonary shunt). OS and PFS were computed with the Kaplan-Meier method and survival distributions were compared with a log-rank test. OS and PFS were presented as median and 95% CIs. Survival curves were drawn according to BCLC class, ALBI grade, C-P class, and scores. Survival analyses in each BCLC class were sub-stratified according to ALBI to investigate whether a more appropriate prognostic stratification could be provided. Survival analyses were sub-stratified according to ALBI also in C-P class A and C-P score 5. The limited number of patients in C-P class B and C-P scores 6–8 did not allow for a reliable sub-stratification of these groups of patients. Further survival analyses were performed based on treatments before TARE, by splitting the population into “naïve” and previously treated patients. Univariate and multivariate Cox proportional hazards regression models were performed to determine the strength of the association between each prognostic factor and OS/PFS. Hazard ratios (HRs) and 95% confidence intervals (CIs) were reported. Significant variables in univariate regression analysis and variables of interest according to literature and guidelines (5) were included in multivariate regression analysis with the backward stepwise method. The best model was identified using the Akaike Information Criterion (AIC). All statistical analyses were performed with STATA (STATA version 17.0 SE, StataCorp LLC, College Station, TX, USA). A *p*-value ≤ 0.05 was considered statistically significant.

## Results

### Baseline patients' characteristics

Seventy-nine consecutive patients with HCC eligible for TARE were screened. Among them, three patients were not treated for a high pulmonary shunt at dosimetry. In one additional case, TARE was contraindicated for a high level of total bilirubin. Among the 75 patients who underwent TARE, three patients lost at follow-up were non-contactable by phone and therefore excluded from the analysis. Finally, data of seventy-two patients were analyzed. Baseline patients' characteristics and treatment details are described in Table 1.

**Table 1:** Baseline patients' characteristics and treatment details.

**Baseline patients' characteristics (*****N*** = **72)**	
**Age, years**	70.76 (65–78)
**Sex, *n* (%)**	
- Males	58 (81)
- Females	14 (19)
**Etiology, *n* (%)**	
- HCV	32 (44)
- HBV	5 (7)
- Alcohol	9 (13)
- Others	26 (36)
**Previous treatments, *n* (%)**	
- No	30 (42)
- Yes	42 (58)
Type of treatment, n*	
- Surgery	19
- TAE/TACE	39
- RF/PEI	5
- Sorafenib	1
**Tumor localization, *n* (%)**	
- Right lobe	19 (26)
- Left lobe	7 (10)
- Bilateral	46 (64)
**Number of lesions, *n* (%)**	
- 1	5 (7)
- 2	5 (7)
- Multiple	62 (86)
**PVT, *n* (%)**	
- No	36 (50)
- Type A	22 (31)
- Type B	14 (19)
**BCLC class, *n* (%)**	
- B	32 (44)
- C	40 (56)
**C-P score, *n* (%)**	
- 5	51 (71)
- 6	12 (16)
- 7	7 (10)
- 8	2 (3)
**ALBI grade, *n* (%)**	
- 1	15 (21)
- 2	51 (71)
- 3	6 (8)
Total bilirubin, mg/dl	0.93 (0.52–1.13)
Albumin, g/l	37.77 (35–40)
AFP, ng/ml	1,455.36 (5–678.6)
**TREATMENT CHARACTERISTICS**	
**Number of procedures, *n* (%)**	
- 1	63 (88)
- 2	9 (12)
Time diagnosis-TARE	33.75 (4.5–52.67)
Administered activity, GBq	0.97 (0.6–1.27)
Tumor absorbed dose, Gy	209.53 (136.5–258)
Pulmonary shunt, %	7.13 (3.55–9)
Volume treated lesion, mL	267.46 (139–316)

N, number of patients; HCV, Hepatitis C Virus; HBV, Hepatitis B Virus; TAE, Transarterial Embolization; TACE, Transarterial Chemoembolization; RF, Radiofrequency; PEI, Percutaneous Ethanol Injection; PVT, portal vein thrombosis; C-P, Child-Pugh; AFP, alpha-fetoprotein; TARE, Transarterial Radioembolization; GBq, Giga-Becquerel; Gy, Gray.

* Twenty-six patients underwent more than one treatment.

### OS analyses

**Figure 1:**
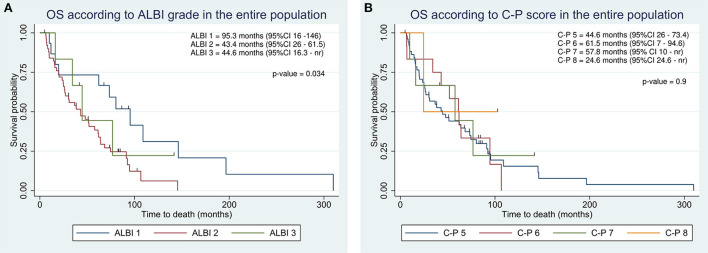
Kaplan-Meier overall survival estimate according to integration of albumin-bilirubin (ALBI) grade **(A)** and Child-Pugh classification **(B)** in the entire population.

At the end of the study, 14 of 72 (19%) patients were still living. The cause of death was related to liver disease in 57 patients (i.e., liver failure or metastatic spread of HCC). One patient died of bronchopneumonia. The median OS of the entire cohort of patients was 51 months. OS in BCLC class B was longer than in class C (62 vs. 27 months; Table 2). Kaplan-Meier curves according to ALBI grade identified categories of patients with the different outcomes (Figure 1A). Specifically, Patients with ALBI 1 showed improved survival as compared to ALBI 2 (95.3 vs. 43.4 months). C-P did not provide a suitable prognostic stratification (Figure 1B).

**Table 2:** Overall survival and progression-free survival analyses in the overall population.

	**OS, median (95% CI)**	***p*** **-value**	**PFS, median (95% CI)**	***p*** **-value**
Total cohort	51.3 (31–68.4)		5.4 (4–9)	
**BCLC class**				
- B	62 (44.6–94.6)	0.236	9 (4.3–11.8)	0.282
- C	27 (16.2–64)		4.4 (2.3–6)	
**ALBI grade**				
- 1	95.3 (16–146)	0.034*	11.8 (2.7–19)	0.128
- 2	43 (26–61.5)		4.8 (2.5–9)	
- 3	44.6 (16.3–nr)		4.3 (2.3–nr)	
**Child-Pugh class**				
- A	51.3 (31–68)	0.53	6 (4,–10)	0.024*
- B	57.8 (9.9–nr)		2.6 (1.6–5.5)	
**Child-Pugh score**				
- 5	44.6 (26–73.4)	0.9	8.8 (4.7–11.5)	0.04*
- 6	61.5 (7–94.6)		2.3 (1.4–13.4)	
- 7	58 (10–nr)		2.6 (1.8–5.5)	
- 8	24.6 (24.6–nr)		1.6 (1.6–nr)	
**Portal vein thrombosis**				
- No	62 (44.6–80.3)	0.24	8.8 (4.3–11.7)	0.569
- Type A	43 (15.3–145.4)		4.8 (2.3–11.3)	
- Type B	16.2 (7.4–26.4)		2.3 (1.6–5.4)	
**Previous treatment**				
- No	19.8 (11.7–30)	<0.001	9 (2.3–11.7)	0.57
- Yes	74.6 (58–94.6)		4.8 (2.6–8.5)	

OS, Overall Survival; CI, Confidence Intervals; PFS, Progression Free Survival.

**Figure 2:**
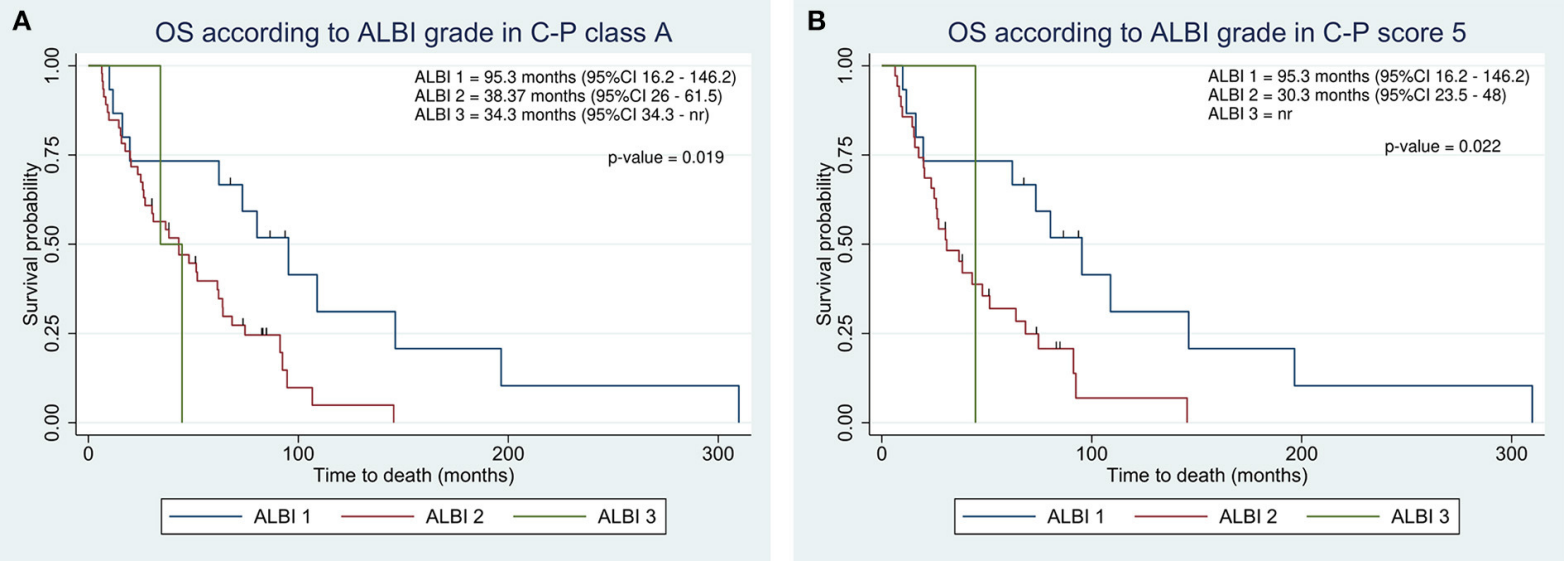
Kaplan-Meier overall survival estimate according to integration of albumin-bilirubin (ALBI) grade within Child-Pugh class A **(A)** and score 5 **(B)**.

Integration of albumin-bilirubin (ALBI) grade identified clusters of patients with different prognoses even within C-P classes. In C-P class A, we observed a progressive decrease in survival in agreement with ALBI (95.3 vs. 38.37 vs. 34.4; Figure 2A). Similarly, C-P score of 5 showed an OS of 95.3 months in ALBI 1 as compared to 30.3 in ALBI 2 (Figure 2B).

**Figure 3:**
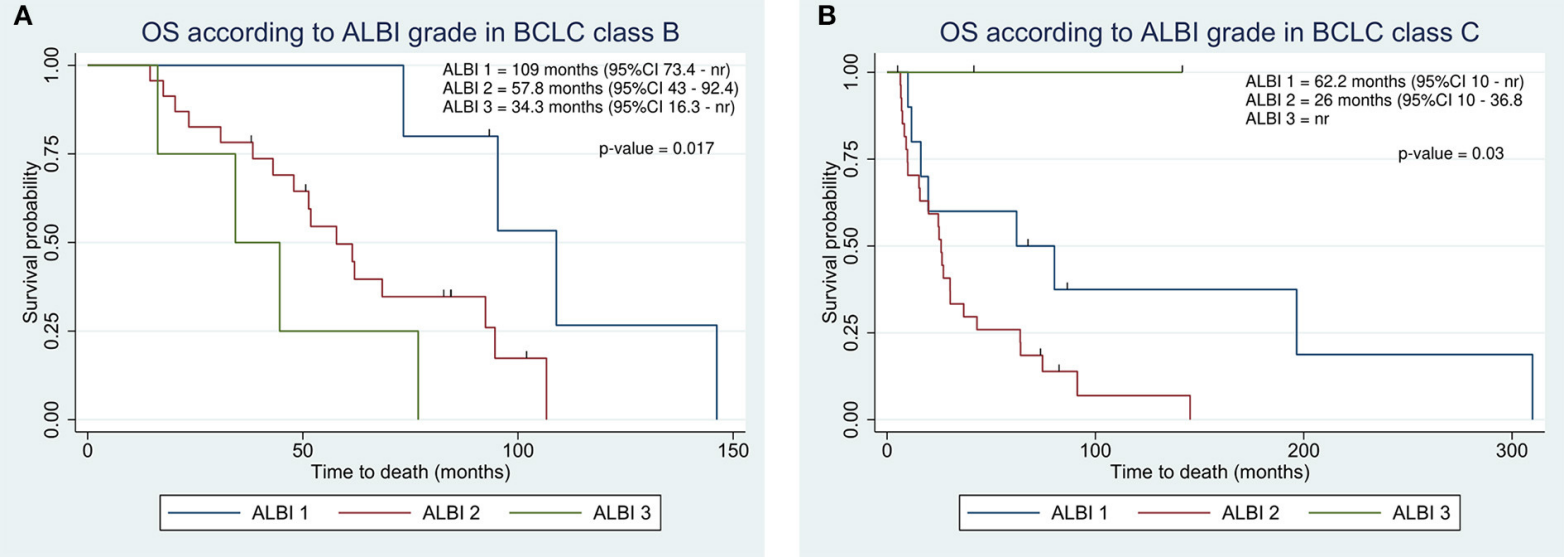
Kaplan-Meier overall survival estimate according to integration of albumin-bilirubin (ALBI) grade within Barcelona Clinic Liver Cancer (BCLC) class B **(A)** and C **(B)**.

We assessed the ability of survival stratification of both ALBI grade and C-P classification within BCLC classes. The ALBI grade revealed a high predictive power in both BCLC classes, resulting in ALBI 1 patients with better prognosis as compared to ALBI 2 and 3 in both BCLC classes (Figures 3A,B). Conversely, the C-P classification did not reach at any significance (*p*-values of 0.98 and 0.5 in BCLC B and C, respectively). In BCLC class B, C-P class A achieved an OS of 62 vs. 58 months in C-P B. In BCLC C, OS in C-P class A was 27 months as compared to 24.6 in C-P class B.

The presence and degree of PVT affected OS in the entire cohort, despite not reaching statistical significance. Patients with no PVT survived longer as compared to patients with Type A and Type B PVT, resulting in 62, 43, and 16 months, respectively (Table 2).

### OS analyses according to previous treatments

**Figure 4:**
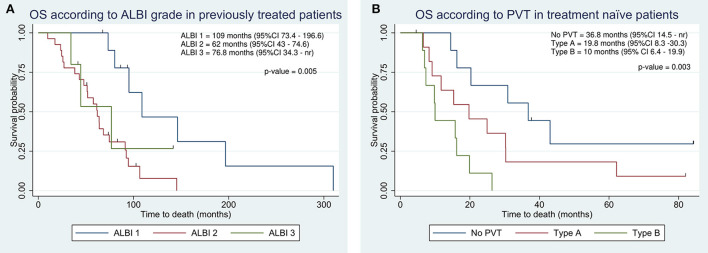
Kaplan-Meier overall survival estimate according to integration of albumin-bilirubin (ALBI) grade in previously treated patients **(A)** and according to portal vein thrombosis (PVT) in treatment naïve patients **(B)**.

In previously treated patients, OS was significantly higher as compared to treatment naïve patients, resulting in 74.6 vs. 19.8 months (Table 2). The ALBI grade provided an effective survival stratification among patients who underwent other treatments before TARE (Figure 4A), whereas treatment naïve patients did not (16.2 vs. 20.3 months, *p*-value = 0.7). In treatment naïve patients, a statistically significant difference in OS was found in the presence and degree of PVT (Figure 4B). Even in this case, C-P classification was not significantly associated with survival in both classes (*p*-values of 0.46 and 0.43).

### PFS analyses

**Figure 5:**
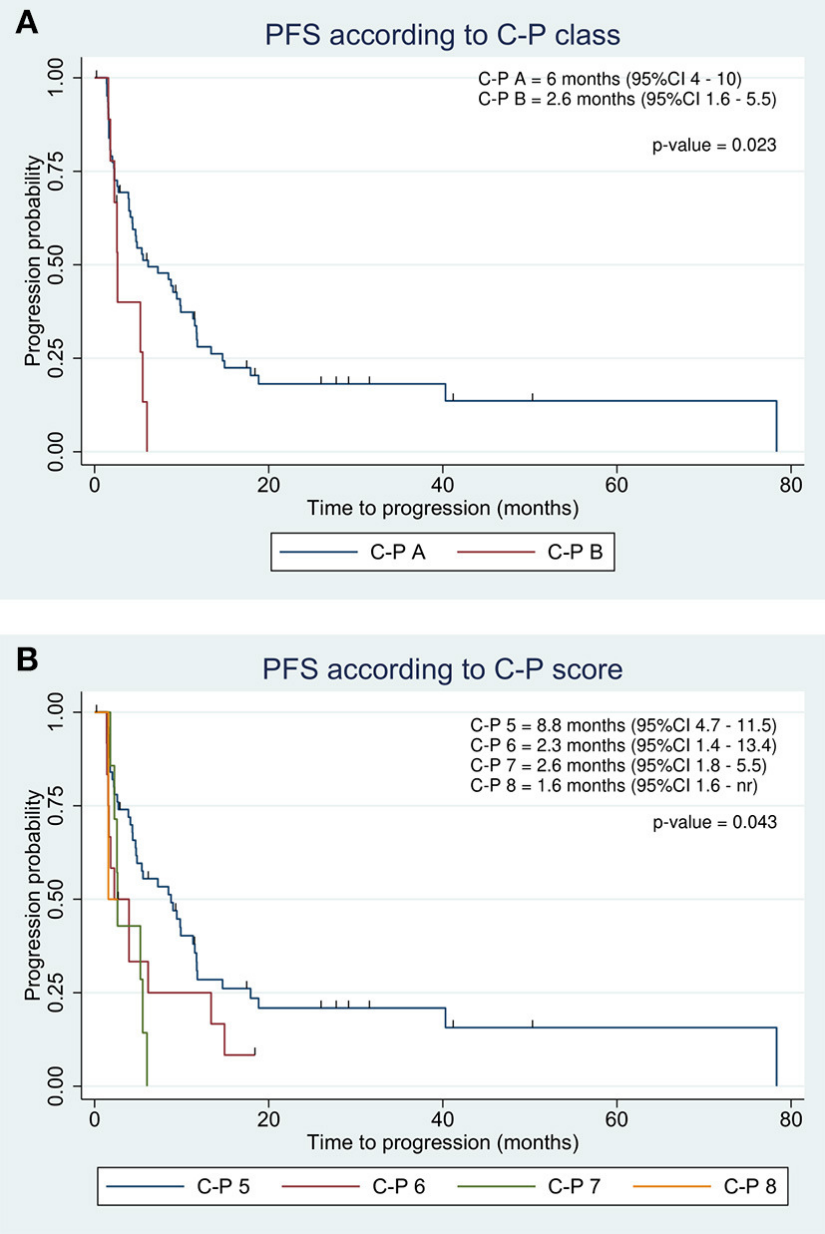
Kaplan-Meier progression-free survival estimate according to C-P class **(A)** and score **(B)** in the entire population.

Two patients showed a complete response at imaging follow-up and did not experience disease progression. The median PFS of the entire cohort was 5.4 months (Table 2). At the end of the study, 58 patients had disease progression. Although the number of patients in progression was almost equal to the number of deaths, the two groups overlapped only partially (61 of 72 matched). ALBI grade was not significantly associated with PFS. However, median PFS was longer in ALBI 1 than in ALBI 2/3 (Table 2). PFS according to the C-P classification resulted significantly improved in the lower classes. Specifically, C-P class A showed a PFS of 6 months as compared to 2.6 months in class B (Figure 5A). Similarly, C-P score 5 exhibited a higher PFS than the other classes, achieving 8.8 months (Figure 5B).

### Survival after TARE

The median survival after TARE in the entire population was 16 months (95% CI 11.7–17.7). The ALBI grade provided a better prognostic stratification as compared to the C-P classification, despite not reaching significance criteria (*p*-values of 0.17 and 0.3, respectively). Patients with ALBI 1 showed a prolonged survival as compared to ALBI 2 and 3 (29 vs. 13.3 vs. 10.8 months). According to the C-P classification, we found the highest survival in score 5 (17 months). However, survival after TARE in C-P score 7 was higher as compared to score 6 (10.8 vs. 6.3 months).

### Univariate and multivariate Cox analyses

Multivariate Cox analysis adjusted for lines of therapy and the elapsed time between diagnosis and TARE showed that Type B PVT, ALBI grade 2, and volume of treated lesions were significantly and independently associated with OS (HR 2.6, 2.43, and 1.001, respectively). Only Hepatitis C Virus (HCV) etiology of liver disease has been identified as a favorable prognostic factor for survival with 0.47 HR (Table 3). C-P scores 5 and 6 and AFP were identified as independent prognostic factors significantly associated with PFS, with HRs of 2.2, 3.15, and 1, respectively (Table 3).

**Table 3:** Univariate and multivariate Cox regression analysis of variables associated with overall survival (OS) and progression-free survival (PFS).

	Overall survival				Progression free survival			
	**Univariate**		**Multivariate**		**Univariate**		**Multivariate**	
	**analysis**		**analysis**		**analysis**		**analysis**	
	**HR (95% CI)**	***p*** **-value**	**HR (95% CI)**	***p*** **-value**	**HR (95% CI)**	***p*** **-value**	**HR (95% CI)**	***p*** **-value**
Age	0.98 (0.96–1.01)	0.34			0.99 (0.97–1.02)	0.87		
Sex	1.28 (0.63–2.62)	0.48			1.2 (0.59–2.45)	0.61		
**Etiology**								
- HCV	0.55 (0.25–0.87)	0.03*	0.47 (0.25–0.87)	0.016*	0.76 (0.45–1.29)	0.3		
- HBV	1 (0.4–2.6)	0.97			1.04 (0.41–2.63)	0.93		
- Alcohol	1 (0.48–2.18)	0.95			0.96 (0.45–2.04)	0.92		
- Metabolic/Other	1.9 (1.1–3.27)	0.02*			1.38 (0.8–2.4)	0.26		
Treatment before TARE (no vs. yes)	0.25 (0.14–0.44)	<0.001*	1.13 (0.52–2.43)	0.755	1.06 (0.8–1.4)	0.7		
- Surgery	0.51 (0.28–0.93)	0.02*			0.92 (0.51–1.66)	0.77		
- TAE/TACE	0.37 (0.21–0.64)	<0.001*			1.06 (0.63–1.8)	0.83		
Disease localization (mono vs. bilobar)	0.5 (0.29–0.86)	0.01*			1.34 (0.77–2.33)	0.29		
Number of lesions (single vs. multifocal)	1.29 (0.55–3.05)	0.54			2 (0.86–4.74)	0.07		
**PVT**								
- No	Reference		Reference		Reference			
- Type A	1.16 (0.63–2)	0.63	1.44 (0.75–2.77)	0.28	1.09 (0.6–2)	0.77		
- Type B	1.8 (0.9–3.7)	0.097	2.6 (1.2–5.6)	0.014*	1.46 (0.7–2.9)	0.29		
Volume of treated lesions	1 (1–1.002)	0.05*	1.001 (1-1.002)	0.013*	1 (0.99–1)	0.64	1 (0.99–1)	0.09
BCLC score (B vs. C)	1.43 (0.84–1.44)	0.18			1.33 (0.79–2.25)	0.28		
**Child–Pugh score**								
- 5	Reference				Reference			
- 6	0.95 (0.47–1.9)	0.88			1.85 (0.94–3.64)	0.07	2.2 (1.06–4.6)	0.035*
- 7	0.8 (0.3–2.3)	0.7			2.65 (1.14–6.2)	0.023*	3.15 (1.3–7.5)	0.010*
- 8	0.5 (0.06–3.65)	0.4			3 (0.4–24)	0.27	4.4 (0.5–35.5)	0.166
Child Pugh class (A vs. B)	0.73 (0.29–1.84)	0.48			2.4 (1.09–5.28)	0.04*		
**ALBI grade**								
- 1	Reference		Reference		Reference			
- 2	2.6 (1.2–2.2)	0.012*	2.43 (1.07–5.5)	0.033*	1.7 (0.85–3.27)	0.135		
- 3	1.6 (0.5–5.25)	0.44	2 (0.58–6.94)	0.274	2.7 (0.97–7.6)	0.057		
Albumin	0.99 (0.94–1.05)	0.86			0.94 (0.89–1)	0.06		
Total bilirubin	1.08 (0.71–1.66)	0.7			1.02 (0.63–1.64)	0.93		
Alpha fetoprotein	1 (0.99–1)	0.77			1.01 (1.01–1.01)	0.03*	1 (1)	0.002*
Tumor absorbed dose	1 (0.99–1)	0.58			1 (0.99–1)	0.79		
Tumor absorbed dose (<150 vs,=. >150 Gy)	1.05 (0.6–1.85)	0.85			1.55 (0.86–2.8)	0.13	1 (0.9–3.2)	0.09
Time diagnosis-treatment	0.96 (0.95–0.98)	<0.001*	0.96 (0.95–0.98)	<0.001*	1 (0.99–1)	0.77		

HR, Hazard Ratio; HCV, Hepatitis C Virus; HBV, Hepatitis B Virus; TAE, Transarterial Embolization; TACE, Transarterial Chemoembolization; PVT, portal vein thrombosis; Gy, Gray.

## Discussion

Our study confirmed that ALBI grade was successful in the prognostic assessment of HCC in patients treated with TARE. Moreover, C-P classification resulted in unreliable in terms of prognosis and ALBI grade outperformed C-P classification across BCLC classes. In our cohort, median OS in ALBI 1 was more than two times higher than patients with ALBI 2, with a survival difference of more than 50 months. Conversely, C-P class A survival resulted lower than class B, as well as a score of 5 as compared to a score of 6. The unreliability of the C-P classification was in contrast with Johnson et al. and Antkowiak et al. who found that the C-P classification allows a fair degree of prognostic discrimination (14, 18).

The ALBI model, developed and tested in HCC and cirrhotic patients, resulted in an excellent prognostic stratification. Clusters of patients with different outcomes were identified according to ALBI within C-P class A, highlighting the great intra-class heterogeneity of patients with HCC. Within C-P class A patients treated with surgical resection, they observed a difference in survival of more than 50 months between ALBI 1 and 2 (14), consistent with our findings. Comparable results are reported in the literature in different clinical settings, such as surgery, transarterial treatments, and sorafenib (12, 19–26).

Antkowiak et al. also demonstrated that ALBI is an effective method for prognosis stratification in a larger population. Median OS resulted in 46.7, 19.1, and 8.8 months in ALBI 1, 2, and 3, respectively (18).

Mohammadi et al. applied the ALBI model to 124 patients with unresectable HCC treated with TARE. The survival difference between ALBI 1 and 2 was 9.9 months, whereas between C-P class A and B was only 4.7 months. In reclassifying C-P A patients with ALBI, the gap between ALBI 1 and ALBI 2 remained at 9.9 months (27). Similarly, in our cohort, the ALBI grade assessed before TARE demonstrated a great power of prognostic discrimination within the C-P classes (Figure 2). In class A, patients with ALBI 1 showed a difference of almost 60 months in survival as compared to ALBI 2 and 3. This result further confirmed the limits of C-P in prognostic stratification and the great heterogeneity within C-P classes, suggesting that these patients might not be considered equally in treatment decisions. Unfortunately, we were unable to apply the same approach in C-P score 6, as 11 of 12 patients were ALBI 2.

Hickey et al. demonstrated the superiority of ALBI in predicting survival even within C-P class B. A survival difference of 4 months was observed between ALBI 2 and ALBI 3 in 428 patients with HCC treated with TARE. However, 215 patients belonged to C-P class B, whereas only 4 patients were assigned to ALBI 1 and therefore almost the whole population was distributed between ALBI 2 and 3 (28). Conversely, the majority of patients treated in our institute were fit and with preserved liver function. Among 72 patients, only nine patients were classified in C-P B, as well as six patients were graded as ALBI 3. Therefore, it was not possible for us to draw any conclusions regarding these two categories.

Even within BCLC classes, the ALBI grade performed better than the C-P classification. In BCLC class B, the ALBI grade recognized a group of patients with a significantly higher prognosis (Figure 3A). However, according to the BCLC classification, patients with C-P class A and B are considered equally in intermediate stages. Therefore, although EASL guidelines currently do not discriminate between preserved and moderately impaired liver function (5), clinicians should also consider the ALBI grade when selecting patients for treatment with TARE. In a *post-hoc* analysis of the Sorafenib vs. Radioembolization in Advanced Hepatocellular Carcinoma (SARAH) trial, Palmer et al. suggested that preserved liver function (ALBI 1) and a tumor burden ≤25% might be useful criteria to identify patients to be treated with TARE (29).

As the majority of HCCs were selected for TARE when other treatments failed, we assumed that the effect of previous surgery or ablative therapies could influence our results. Therefore, we split the population into naïve and previously treated patients, and we performed survival analyses on the two populations separately. OS in naïve patients was about 4-fold shorter than in previously treated patients (19.8 vs. 74.6 months) and in this group of patients, the ALBI grade was not correlated with prognosis, supporting that the ALBI grade has a prognostic value only in a specific setting of patients.

We also found a significant difference in terms of survival according to PVT, supporting the assumption that tumor extension—over liver function—had a greater impact on prognosis in this subset (Figure 4B).

Therefore, considering that patients with HCC are treated with TARE in the first line when ineligible for other therapies as in case of advanced disease and in presence of PVT (7, 30) that does not represent a contraindication for TARE, it should be considered that the presence and extent of PVT negatively impact on prognosis (7, 31, 32), as further confirmed by our results. Several classification systems of PVT are available, and we selected Xu classification for our study, as it demonstrated a prognostic impact in a simple and clear fashion (16, 33). Nevertheless, we proved that naïve patients benefitted from TARE (median OS of 19.8 months) regardless of the presence and the extent of PVT (OS rates in patients with no, Type A, or Type B PVT were 36.8, 19.8, and 10 months, respectively), compared to untreated patients with HCC, who survive on average 6.8 months (4). Notably, the median survival rate after TARE was almost comparable to that reported from CIRT—the largest prospective European study on a study on TARE—(16 vs. 16.5 months) (34).

## Conclusions

In conclusion, the ALBI grade allowed an effective prognostic stratification in patients with intermediate-advanced HCC treated with TARE. Moreover, C-P classification resulted in unreliable in terms of outcome prediction. However, the ALBI grade is applicable only to a specific subset of patients. In cases with HCC not treatable by surgery or ablative therapies, not only the ALBI grade but rather disease-related features, such as disease burden and PVT degree, should be carefully considered before performing TARE. Our results suggested that current guidelines might be improved by replacing the C-P classification with the ALBI grade for a better treatment decision-making.

## Limitations

Our study was affected by some limitations related to its retrospective nature and the relatively small sample size. It was not possible to comprehensively evaluate and therefore draw conclusions about patients in advanced classes, such as ALBI 3 and C-P B. Our cohort was rather unbalanced, as most patients fell into ALBI 1 and 2 and C-P A, restricting the analysis to these classes. The survival estimate was partially limited by the presence of a proportion of censored patients (19%). OS might be overestimated for patients with disease progression and forwarded to ADI at the last follow-up as we assumed a 4-month survival time. Finally, in the absence of liver disease burden quantification, we used the volume of treated lesions calculated at pre-treatment scintigraphy as a surrogate.

## Data availability statement

The raw data supporting the conclusions of this article will be made available by the authors, without undue reservation.

## Ethics statement

The Ethics Committee of IRCCS Humanitas Research Hospital approved the study and did not require a written consent to participate due to the observational and retrospective nature of the study. All patients signed a written informed consent for the diagnostic and therapeutic procedures described in the study.

## Author contributions

FG and MR conceptualized and designed the study. MR, FG, CP, RZ, and GT collected the clinical data. FG and AA performed data analysis. FG, CP, and MS prepared the tables and figures. FG and MS interpreted the analyses results and drafted the paper. MS and AC critically commented the paper. All authors critically revised the paper and approved the submitted version of the manuscript. All authors contributed to the article and approved the submitted version.

## Conflict of interest

AC: AmGen, speaker honorarium; Blue Earth Diagnostics, advisory board, speaker honorarium; Novartis, Advisory Board, speaker honorarium; Sirtex, speaker honorarium. The remaining authors declare that the research was conducted in the absence of any commercial or financial relationships that could be construed as a potential conflict of interest.

## Publisher's note

All claims expressed in this article are solely those of the author and do not necessarily represent those of their affiliated organizations, or those of the publisher, the editors and the reviewers. Any product that may be evaluated in this article, or claim that may be made by its manufacturer, is not guaranteed or endorsed by the publisher.
